# Megagametophyte maturation dynamics and flavonol changes during *Arbutus unedo* flower development

**DOI:** 10.3389/fpls.2025.1694629

**Published:** 2025-11-06

**Authors:** Alessia D’Agostino, Gabriele Di Marco, Antonella Canini, Angelo Gismondi

**Affiliations:** Laboratory of Botany, Department of Biology, University of Rome “Tor Vergata”, Rome, Italy

**Keywords:** strawberry tree, flowering, secondary metabolites, DPBA staining, flavonols, metabolomics, histological analyses

## Abstract

The flowering rhythm of *Arbutus unedo* L. (strawberry tree) is unusually prolonged and remains largely unexplored. Thus, this study characterizes its floral development through seven defined stages (St1-St7), from meristematic buds to anthesis. Histological analyses revealed that anther differentiation occurred earlier than ovule development, which displayed a prolonged apparent slowdown. To understand whether phytochemicals could play a signaling role in this context, due to their potential effects on hormones, proteins and DNA, total and specific quantitation of these compounds was performed by spectrophotometry and targeted (HPLC-DAD) and untargeted (GC-MS) metabolomic approaches. A general decline in secondary metabolite levels was observed from St1 to St7, except for flavonols, which exhibited a non-linear accumulation pattern. These findings were corroborated by principal component analysis and qPCR assays of genes involved in phenylpropanoid and flavonoid biosynthesis. Fluorescence histochemistry demonstrated that flavonols (kaempferol and quercetin) were synthesized with a stage- and tissue-specific localization, particularly at St2, St4, and St7, and accumulated mainly in the epidermis, pollen, and ovules. Their subcellular localization varied across stages, with notable nuclear accumulation in ovary and ovule tissues, suggesting a potential role in transcriptional regulation. In conclusion, the developmental dynamics of the megagametophyte and the spatiotemporal distribution of flavonols seem to influence flower maturation in *A. unedo*, indicating a possible regulatory function for these compounds beyond their conventional roles.

## Introduction

*Arbutus unedo* L., known as strawberry tree, is an evergreen woody fruit species belonging to the Ericaceae family with a circum-Mediterranean natural distribution (Bebek Markovinović et al., 2022). This plant has considerable ecological relevance in southern European forests due to its resilience toward abiotic and biotic environmental stressors. *A. unedo*, with its notable phenotypic plasticity and evolutionary adaptability, is expected to be a key component in the Mediterranean flora’s resilience to future climate change (Almeida et al., 2022; Martins et al., 2022). Strawberry tree holds economic value due to its fruits and leaves, which contain bioactive compounds (sugars, fibers, vitamins) with medicinal properties like antiseptic, diuretic, laxative, and anti-inflammatory effects (Šic Žlabur et al., 2020; Martins et al., 2022).

Many works have been devoted at characterizing strawberry tree berries and leaves, especially as raw material for the production of sustainable functional foods and nutraceutical supplements (Bebek Markovinović et al., 2022), while structure and development of the flower in this species still remain under-investigated topics (Villa, 1982, 1983; Martins et al., 2021, 2022), although they are unique in terms of architecture and timing of production. Notably, *A. unedo* undergoes a slow phenological cycle, as the full development from floral buds to mature fruits takes close to 18-20 months. About 8-12 months are required to produce complete, bell-shaped, sympetalous, and pinkish-white flowers; thus, fruit ripening occurs simultaneously with the next bloom in Autumn (i.e., flowers and fruits can be found simultaneously on the same tree). The literature describes three distinct stages: flower buds, flowers at anthesis, and fruit development (Villa, 1982, 1983). Particular attention was given to the prolonged quiescence of the flower buds, which emerge in June and remain dormant for several months. In contrast, anthesis occurs from October-November and can last until January. Other members of the Ericaceae family, such as blueberry (*Vaccinium corymbosum* L.) and rhododendron (*Rhododendron* spp. L.), complete flower development in considerably shorter ontogenetic time frames (Bozek, 2009; Nowaczyk and Krzymińska-Bródka, 2022). This difference underscores the uniqueness of *A. unedo* reproductive schedule and highlights the need to investigate the anatomical, morphological, and metabolic events regulating its flower development.

The molecular events driving flower development are spatially and temporally controlled and are associated with significant metabolic changes, such as the production of secondary metabolites (Önder et al., 2022). Flowering is a finely regulated biological process essential for plant ecology, reproduction, and evolution. Among the different classes of secondary metabolites present in flowers, flavonols were selected as the main focus because of their well-documented involvement in reproductive development. In fact, beyond their canonical functions in UV protection and antioxidant defense, flavonols have been reported to play key roles in male fertility, pollen germination, stigma receptivity, and pollen-stigma interactions (Mo et al., 1992; Ylstra et al., 1992; Besseau et al., 2007; Wang et al., 2024). Furthermore, their ability to interact with hormonal pathways, particularly auxin transport, suggests a regulatory role in the coordination of reproductive organ differentiation (Lewis et al., 2011; Luo et al., 2016). Based on this evidence, molecular profiling across seven flower developmental stages was performed to investigate the functional contribution of flavonols within reproductive tissues.

## Materials and methods

### Plant material

*A. unedo* L. flowers were randomly collected from 3 different strawberry trees which are about 30 years old and grow in the same area of the Botanical Gardens of Rome “Tor Vergata” (Rome, Italy; Lat: 41.84758, Long: 12.64385). The sampling was carried out between June 2023 and January 2024, allowing to distinguish seven different developmental stages starting from immature flower buds (Stage 1) to fully open flowers (Stage 7), according to significant morphological changes and the dimensions reported in **Table 1**. For each stage, 300 flowers were collected (voucher codes: ARB1-21) and used to carry out the whole set of experiments. Once collected, the plant material was transported in refrigerated containers to the Laboratory of Botany of the Department of Biology at the University of Rome Tor Vergata, screened for absence of mechanical damage and uniformity, and directly subjected to the adequate processing for the morphological, genetic and biochemical investigations. In detail, a portion was used fresh while the remaining one was powdered with pestle and mortar in the presence of liquid nitrogen and kept at −80 °C until the analysis. The authors state that the material used for the present research has been collected in accordance with the principles of ethics reported in the IUCN Policy Statement.

**Table 1:** Morphometric data from *A. unedo* flowers. Mean value of dimension (in mm) for the flower elements collected at each stage and related standard error are shown.

Flower elements and relative parameters		Flower developmental stages						
		1	2	3	4	5	6	7
Entire flower structure	length	0.57 ± 0.02	2.1 ± 0.1	3.6 ± 0.2	5.4 ± 0.19	7.5 ± 0.2	8.4 ± 0.23	9 ± 0.26
	width (max diameter)	0.5 ± 0.01	2.5 ± 0.09	3 ± 0.1	3.3 ± 0.12	4.5 ± 0.1	6 ± 0.3	7.7 ± 0.19
	aperture	–	–	–	–	–	–	4.1 ± 0.3
Gynoecium	pistil length	–	1.6 ± 0.1	3.3 ± 0.16	4.5 ± 0.1	7 ± 0.14	8 ± 0.25	8.8 ± 0.17
	ovary width (max diameter)	–	1 ± 0.03	2 ± 0.05	2 ± 0.05	2.2 ± 0.1	2.5 ± 0.1	2.5 ± 0.2
	style length	–	–	1.7 ± 0.1	2.3 ± 0.12	3.2 ± 0.1	4.5 ± 0.2	5 ± 0.2
	protrusions thickness	–	–	0.1 ± 0.04	0.15 ± 0.01	0.17 ± 0.01	0.19 ± 0.01	0.3 ± 0.1
Androecium	anther length	–	–	1.8 ± 0.1	2 ± 0.09	2 ± 0.1	2 ± 0.05	2 ± 0.02
	anther width	–	–	0.5 ± 0.03	0.54 ± 0.02	0.6 ± 0.06	0.62 ± 0.04	0.7 ± 0.01

### Morphological, histological and histochemical analyses

Pictures of fresh samples were captured through a stereomicroscope (Leica ZOOM 2000). For the fixation, samples were rinsed in water twice, gently dried on paper tissue and immersed in 4% (w/v) paraformaldehyde in 0.1 M phosphate saline buffer pH 7.2 (PBS) under mild vacuum condition overnight at 4 °C. Then, they were washed twice with PBS (30 minutes each) and dehydrated employing a graded ethanol series (80% and 90% 1 h each step, followed by two 95% ethanol stages 1.5 h each, and finally two passages in 100% ethanol 10 min each). After the last step, ethanol 100% was replaced with Histoclear reagent (Electron Microscopy Sciences) at room temperature and then embedded overnight in paraffin wax (Paraplast, Leica Biosystems) at 57°C. Serial sections of 10 µm-thick were cut on a rotary microtome. Slides were deparaffinized in Histoclear reagent, rehydrated with an ethanol series (from 100% to 70%) and maintained in PBS to be stained with 2.5 mg/mL diphenylboric acid-2-aminoethyl ester (DPBA) reagent and 0.005% (v/v) Triton X-100, according to the previous methods (Muhlemann et al., 2018; Nguyen, 2020), to obtain an *in-situ* fluorescent staining for flavonols. Microscopy detection was carried out by Olympus (Evident) FV4000 Confocal Laser Scanning Microscope after excitation at 458 nm. Kaempferol emission was collected between 472 and 499 nm, while quercetin was monitored applying a filter starting from 585 and arriving to 619 nm. Basal images were captured under bright field. Additionally, the samples were subjected to a general anatomical observation and measurements using an optical microscope equipped by polarized filter (Nikon Eclipse E100).

### Content of phenolic metabolites

A total of 150 mg of plant powder were resuspended in 1.5 mL of methanol:water (50:50, v/v) for 24 h, under agitation, in the dark at 4 °C. After centrifugation at 12.000 g for 10 min, the supernatant was used to carry out the quantitation of phenolic secondary metabolites, as reported below.

Total phenols were estimated by the Folin-Ciocalteu spectrophotometric assay. Briefly, 250 µL of Folin-Ciocalteu reagent (1:10, v/v) and 200 µL of 0.7 M sodium carbonate were added to the flower extract (50 µL). The solution was incubated 2 h in the dark at room temperature and the relative absorbance was read at 760 nm by a microplate reader (Sunrise, Tecan). A calibration curve using increasing amounts of gallic acid was employed as reference. The results were expressed as mg of gallic acid equivalents (GAE) per g of sample fresh weight (mg GAE/g FW).

For flavonoid determination, the aluminium chloride colorimetric method was used. One hundred μL of flower extract was mixed with 20 μL of 10% aluminium chloride (AlCl_3_), 20 μL of 1 M potassium acetate (CH_3_CO_2_K), 300 μL of methanol and 560 μL of ddH_2_O. The sample was incubated for 30 min in the dark at room temperature and the absorbance of the solution was read at 415 nm by a microplate reader. Total flavonoid content was obtained using a calibration curve with increasing concentrations of quercetin and results were expressed as mg of quercetin equivalent (QE) per g of sample fresh weight (mg QE/g FW). The determination of total anthocyanins by spectrophotometric analysis was performed according to the Giusti and Wrolstad method (2001). Briefly, two buffers (0.025 M potassium chloride buffer pH 1.0 and 0.4 M sodium acetate buffer pH 4.5) were prepared and 100 µL of each of them were separately mixed with 25 µL of flower extract. The two samples were incubated in the dark for 15 min; then, the absorbance of both solutions was measured at 530 nm and 700 nm (as background) by a Varian Cary 50 Bio UV-Vis spectrophotometer (GEMINI Lab sustainable equipment, Apeldoorn, The Netherlands). The following formula was used for measuring the absorbance of the sample:


$$\text{A}=\left[{\left({\text{A}}_{x}-{\text{A}}_{700}\right)}_{\text{pH }1.0}-{\left({\text{A}}_{x}-{\text{A}}_{700}\right)}_{\text{pH }4.5}\right]$$

The content of anthocyanins was calculated using the equation reported below:


Anthocyanins=(A×MW×dilution factor×1000)/(ϵ×0.6)

where A is the absorbance measured for the sample at the wavelength, MW is the molecular weight of the cyanidin-3-glucoside as standard equivalent for the quantitation of the total anthocyanin, ϵ is the molar absorptivity of the anthocyanin (i.e., cyanidin-3-glucoside, 26.900), and 0.6 cm is the length of the cuvette employed for the realization of the test. Results were reported as ng of cyanidin-3-glucoside equivalent per mg of sample fresh weight (ng Cy3GE/mg FW).

Lastly, the content of tannins was measured spectrophotometrically (510 nm) according to the protocol of Weidner et al. (2009), following the modifications described in Impei et al. (2015). In particular, a standard calibration curve has been developed with pure catechin (C; 0–300 mg/L; R^2^ = 0.9891) and the total tannins was expressed as µg of catechin equivalent per mg of sample fresh weight (µg CE/mg FW).

Total flavonols were measured following the spectrophotometric assay applied by Jimoh et al. (2010). A total of 50 mg of plant powder were macerated in 0.5 mL of methanol:water (4:1, v/v) for 5 h, under continuous shaking, in the dark at room temperature. After centrifugation at 13.000 g for 10 min, the supernatant (250 µL) was mixed with 250 µL of 2% AlCl_3_ and 250 µL of 5% sodium acetate (CH_3_COONa). The solution was incubated for 2.5 h and then the absorbance was read at 440 nm by a microplate reader (Sunrise, Tecan). A calibration curve, based on increasing amounts of quercetin, was used as reference. The results were expressed as µg of quercetin equivalent (QE) per mg of sample fresh weight (µg QE/mg FW).

### High pressure liquid chromatography analysis

To measure the content of specific phenolic compounds in each extract, an HPLC system was employed. The instrument was provided with a CBM-20A controller, an LC-20 AD pump, a SIL-20a HT autosampler, and an SPDM20A diode array detector (DAD) (Shimadzu, Kyoto, Japan). A Luna 3u C18(2) column (150 mm × 4.60 mm × 3 µm) (Phenomenex, Torrance, CA, USA) and two mobile phases, consisting of 1% formic acid (v/v) (phase A) and methanol (phase B) at a flow rate of 0.95 mL per minute, were used for the chromatographic separation. Column temperature was set at 40 °C, while the elution gradient was set as follows: it began at 15% solvent B and remained constant for 20 min; then, solvent B was linearly increased up to 35% in 20 min and up to 90% in 55 min. At 70 min, the solvent B was reported at the initial condition. LAB-SOLUTION software (Shimadzu) was used for data acquisition. Flavonoid compounds (i.e., resveratrol; quercetin-3-glucoside; myricetin; quercetin; genistein; kaempferol; chrysin; epicatechin) and other phenolics (i.e., gallic acid; 3-hydroxytyrosol; vanillic acid; rosmarinic acid; 4-hydroxybenzoic acid; chlorogenic acid; caffeic acid; syringic acid; *ρ*-coumaric acid; salicylic acid; 1,1-dimethylallyl caffeate; caffeic acid phenethyl ester; 5,7-dimethoxycoumarin) were detected in the extracts at 280 nm. Each metabolite was analyzed in quantitative terms, comparing their retention times (minutes), absorption spectra, and peak areas with those of pure standard molecules (Sigma-Aldrich, Milan, Italy). Results were reported as mg of standard equivalent per g of sample weight (mg SE/g FW).

### Total content of terpenoids

Total terpenoid content was measured following the method of Ghorai et al. (2012). This spectrophotometric assay was performed starting from 250 mg of plant material which were resuspended in 500 µL of ice-cold 95% methanol. The mixture was incubated at room temperature for 48 h in dark. After centrifugation, the supernatant (200 µL) was mixed with 750 µL of chloroform and 50 µL sulfuric acid and incubated for 2 h in the dark. The reddish-brown precipitate was resuspended in 200 µL of 95% methanol. The absorbance was read at 538 nm with a microplate reader (Sunrise, Tecan). As reference, a standard curve with linalool was used. Results were expressed as ng of linalool equivalent (LE) per mg of sample fresh weight (ng LE/mg FW).

### Quantitation of carotenoids

A total of 200 mg of plant powder were resuspended in 1 mL of dichloromethane:methanol (2:1, v/v) for 24 h, under agitation, in the dark at 4 °C. After centrifugation at 12.000 g for 10 min, the supernatant was used to carry out the quantitation according to the protocol of Lichtenthaler (1987). The absorbance of the flower extracts was measured at 470, 655.20, and 653.40 nm by a UV/Vis spectrophotometer (Varian Cary 50 Bio UV–Vis, The Netherlands). Carotenoid levels were calculated by the application of the following formula:


$$\text{Carotenoids}=(1000*{\text{Abs}}_{470}–2.66*{\text{Abs}}_{655.20}–6.38*{\text{Abs}}_{653.40})/126$$

Results were expressed as ng of carotenoids per mg of fresh weight (ng/mg FW).

### Determination of soluble solid content

Plant samples were homogenized (10 mg) and resuspended in 200 µL of distilled H_2_O. After 30 minutes in agitation, the mixture was centrifuged at 10.000 g for 10 min. The supernatant was analyzed with a digital refractometer (model HI96800; Hanna, Woonsocket, RI, USA) to measure the total content of soluble solids (e.g., sugar content). The instrument was calibrated by using 100 µL of distilled water at 20 °C and the results were expressed as µg of soluble sugar equivalents (SSE) per mg of sample fresh weight (µg SSE/mg FW).

### Extraction and quantitation of total proteins

Protein crude extracts were obtained by resuspending 500 mg of a plant powder in 600 µL of cold extraction buffer (25 mM Tris-HCl pH 7.5, 150 mM NaCl, 0.1% Triton X-100, 1 mM EDTA, 5 mM dithiothreitol, and 1X protease inhibitors cocktail). The samples were incubated for 15 min on ice, and centrifuged at 10.000 g for 10 min at 4 °C. The supernatant was recovered and centrifuged again at 13.000 g per 30 min at 4 °C. The novel supernatant was subjected to protein quantitation by the Bradford method (absorbance 595 nm) using a microplate reader (Sunrise, Tecan). Results were expressed as ng of proteins per mg of sample fresh weight (ng/mg FW).

### Gas chromatography-mass spectrometry analysis

The lipophilic fraction of the samples was investigated by GC-MS, according to D’Agostino et al. (2024). One hundred milligrams of fresh plant sample were dissolved in 1 mL of extraction solvent (methanol:chloroform, 50:50; v/v) for 24 h, under agitation, in the dark at 4 °C. After centrifugation at 10.000 g for 8 min, the supernatant was collected and dried out in a speed-vac system (Eppendorf AG 22331 Hamburg, Concentration Plus). The pellet was resuspended in pure methanol:chloroform and subjected to GC-MS. Two microliters of extract were injected in a QP2010 system (Shimadzu, Kyoto, Japan) equipped with an SH-Rtx5MS column (Shimadzu; length 30 m × diameter 0.25 mm × thickness 0.25 µm), in splitless modality. The temperature gradient was configured as follows: an initial step at 50 °C for 2 min, then 200 °C for 2 min (reached by a rate of 3 °C/min), and 300 °C for 5 min (reached by a rate of 4 °C/min). Helium was used as carrier gas at a constant flow of 1 mL/min. For mass spectrometry, the electron impact was set at 70 eV (scanning from 100 to 700 m/z), the ion source temperature at 250 °C, the interface temperature at 280 °C, and the solvent cut time at 6 min. In the chromatograms, all peaks were analyzed. Each analyte was recognized by comparing its mass spectrometry-based molecular profile with those of pure standards registered in the NIST (National Institute of Standard and Technology) Library 14 loaded in detection software of the instrument. The identity of each molecule with the library standards was considered acceptable only for similarity values higher than 85%.

### RNA extraction, cDNA synthesis and quantitative real-time PCR analysis

WizPrep Total RNA Kit (Wizbiosolutions, Loco Hills, United States) was used for total RNA extraction from flower samples, according to the manufacturer’s guidelines. RNA was quantified by a NanoDrop ND1000 spectrophotometer (NanoDrop Technologies) and stored at -80 °C until use. cDNA was synthesized starting from 2.5 µg of RNA with the WizScriptTM cDNA Synthesis Kit (High Capacity). Briefly, reaction buffer (10X), 20X dNTP mix, random hexamer (50 pmol), 200 U WizScript RTase, 40 U RNA inhibitor, and RNase-free water (final volume of 20 μL) were added to RNA and incubated for 2 h at 37 °C and then at 85 °C for 5 min. Finally, cDNA was quantified and stored at -80 °C until the analysis. qPCR reactions were carried out in 20 µL of volume by mixing 50 ng of cDNA, 2X SYBR Green PCR Master Mix (Perkin-Elmer Applied Biosystems, Waltham, MA, USA), and 5 pmol of forward and reverse primers. The primers used for qPCR assays were reported in **Supporting Information 1** and allowed the analysis of the following transcripts: *Anthocyanidin reductase* (ANR), *Anthocyanidin synthase* (ANS), *Chalcone isomerase* (CHI), *Chalcone synthase 1* (CHS1), *Chalcone synthase 2* (CHS2), *Chalcone synthase 3* (CHS3), *Chalcone synthase 5* (CHS5), *Flavanone 3β-hydroxylase* (FHT), *Flavonol synthase* (FLS), *Leucoanthocyanidin reductase* (LAR), *Phenylalanine ammonia-lyase* (PAL). β-*Actin* was employed as housekeeping gene for checking quantity and quality of cDNA templates. The experiments were carried out by a StepOnePlus Real-Time PCR System (Perkin-Elmer Applied Biosystems) set as reported here: 10 min at 95 °C, followed by 45 cycles of 20 s at 95 °C, 30 s at 59 °C. The melting curve, instead, was carried out according to the following conditions: 95 °C for 15 s, 60 °C for 1 min and then, with a rate of 0.3 °C every 15 s, 95 °C were reached and maintained for 15 s. The gene expression of a sample was calculated using the 2^−ΔΔCt^ formula, where the threshold cycle value (Ct) of the target genes was normalized with the Ct of the internal reference control (β-*Actin*) and then to the respective value from the reference sample (ΔΔ^Ct^); in addition, the validation of the method was also performed by Δ^Ct^ variation analysis at different template concentration (Livak and Schmittgen, 2001). Results were represented as mean ± standard deviation (SD) and expressed in Log_10_ with respect to the St1 considered as unit (100).

### Statistical analysis

Three independent biological replicates were performed for each experimental point, and each type of analysis was carried out at least in form of three independent technical replicates (each one representing a mix of several flower units). Data were subjected to one-way analysis of variance (ANOVA) and the differences were evaluated by the *post-hoc* lowest standard deviations (LSD) test. Hierarchical clustering and relative heatmap were generate by an heatmapper tool (http://www.heatmapper.ca/). The relationships existing among the data were estimated by Pearson’s correlation coefficients calculated using SRplot software (Tang et al., 2023). Principal Component Analysis (PCA) was also performed by PCA calculator (Statistics Kingdom, 2017), to reduce the dimensionality of the datasets. In detail, the two principal components with Eigenvalues greater than one were employed to generate the scatter plot.

## Results and discussion

**Figure 1:**
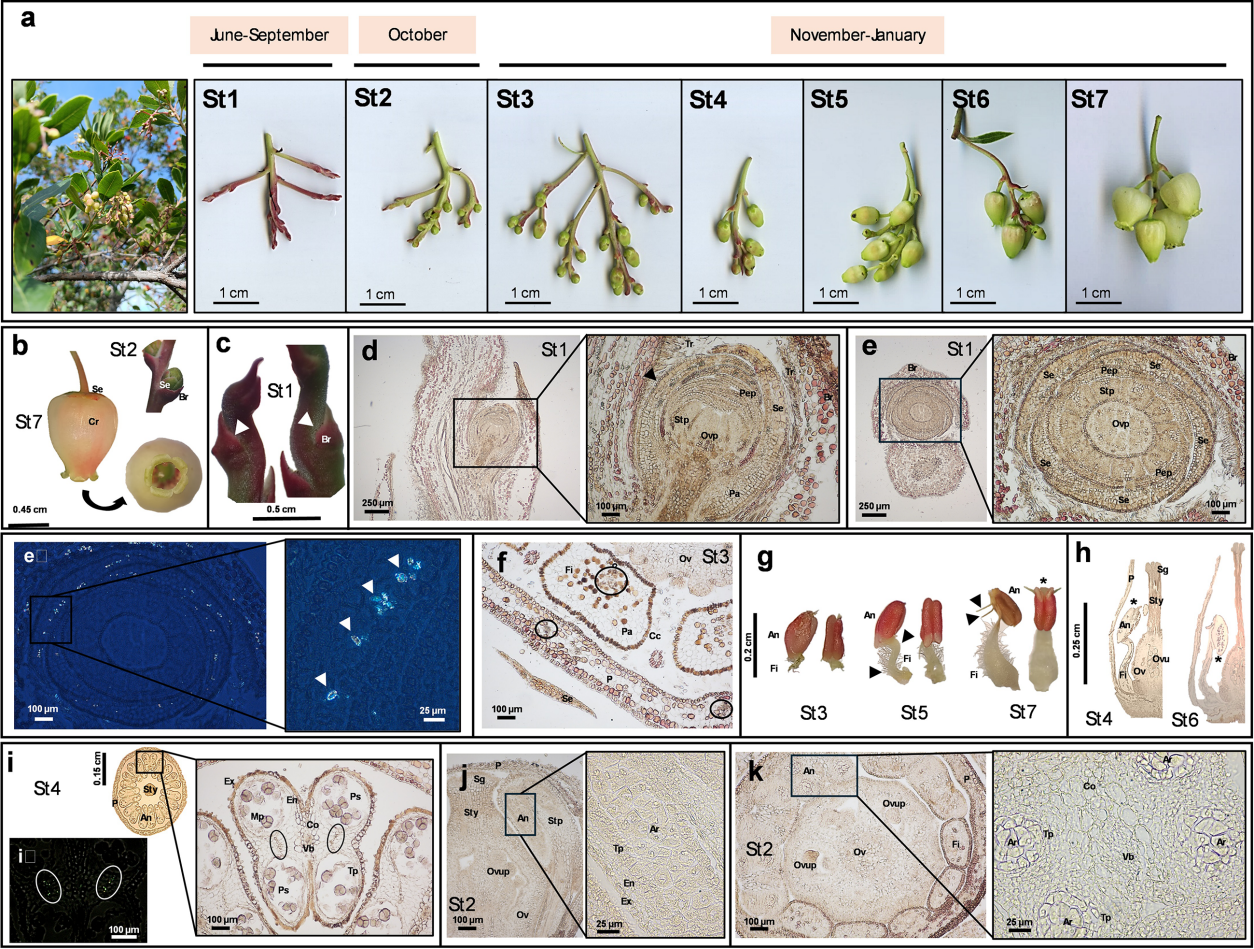
Anatomical images and histological sections from *Arbutus unedo* flower. The representative images reported in the figure were obtained by digital camera (panel **a**), stereomicroscope (panels **a–c**, **g**) or optic microscope (panels d-f and h-k). The dimension bar is reported for each panel. **(a)** Developmental stages (St) of strawberry tree flowers from early buds (St1) to anthesis (St7) and related time frame (upper bar). At the beginning of the series, an image of flowers on the tree is also reported. **(b)** Perianth of flower bud at St2 and of the fully opened flower at St7, showing a green-red pentamerous calyx and an urceolate and sympetalous corolla with 5 short revolute lobes. **(c)** Bulges (white arrows) located on the periphery of the inflorescence apex at St1 and protected by extrafloral bracts. **(d)** Primordium of flower bud at St1 in longitudinal section and relative magnification showing details of its internal structure (the black arrow indicates the monolayer epidermis). **(e)** Primordia of flower bud at St1 in cross section and relative magnification showing details of its internal structure; panel e_2_ represents the same image under polarized light and relative magnification (white arrows indicate calcium oxalate crystals). **(f)** Cross section showing of a petal and filaments at St3 (black circles enclose vascular bundles). **(g)** Morphology of stamens at St3, St5 and St7. Lateral and anterior side for each of them is shown (black arrows indicate the filament curvatures at St5 and two spurs at St7; the asterisk indicates the terminal pores used for pollen release). A dense layer of white hairs can be observed on the filaments. **(h)** Longitudinal sections representing the inversion event of the anthers from St4 to St6 (asterisks indicate the same far end). **(i)** Cross section of flower at St4 and relative magnification of anthers (black circles include some druses, which can be easily detected under polarized light within white circles in the image i_2_). **(j)** Longitudinal section of flower at St2 and relative magnification showing the archesporium in the anther primordium. **(k)** Transverse section of flower at St2 with magnification of the anther primordium. (Legend: An, anther; Ar, archesporium; Br, extrafloral bracts; Cc, cuboid cells; Co, connective; Cr, corolla; En, endotecium; Ex, exothecium; Fi, filament; Mp, mature pollen; Ov, ovary; Ovp, ovary primordium; Ovu, ovule; Ovup, ovule primordium; P, petal; Pep, petal primordium; Se, sepal; Stp, stamen primordium; Sg, stigma; Sty, style; Pa, parenchyma; Ps, pollen sac; Tp, tapetum; Tr: trichome; Vb, vascular bundle).

Species belonging to the genus *Arbutus* (e.g., *A. xalapensis* Kunth*, A. pavarii* Pamp.*, A. canariensis* Veill. ex Duhamel) possess flowers with similar morphology, ecological function and reproductive value, which ensure the evolutionary success of these plants. To date, limited anatomical information is available for the flowers of *A. andrachne* L. (Gökbayrak and Engin, 2021) and *A. unedo* (Villa, 1982, 1983; Simpson, 2010; Martins et al., 2021). This study captured and described the morphological and structural features of *A. unedo* flowers at various maturation stages to better characterize their reproductive elements, which develop much more slowly than in other species (e.g., *Robinia pseudoacacia*, *Tilia cordata*) (Konarska, 2013; Carl et al., 2017). This unusual phenomenon would suggest the existence of peculiar molecular signals able to induce and/or to maintain this condition of biological delay. Although the genetic mechanisms controlling flowering are well understood (Theissen and Melzer, 2007; Ó'Maoiléidigh et al., 2014), the precise timing of sexual organ development remains largely unknown. Recent studies show that secondary metabolites like flavonoids can act as intracellular messengers (Brunetti et al., 2018). This suggests new roles for phytochemicals in plants. Thus, we analyzed their biochemical changes across seven flower stages (St1–7; **Figure 1a**) to explore their potential regulatory function in *A. unedo* blooming.

### Histological investigations reveal a slow development of the gynoecium

Strawberry tree produces complete, bell-shaped, actinomorphic, and pinkish-white flowers, hanging in panicles (20-30 units). The inflorescence usually originates in June from terminal meristems of young stems, where immature buds remain closed within sepals through summer months (St1). After this period of apparent immobility, a slow process of development occurs (October-January), to reach the condition of fully developed flowers (St7) (**Figure 1a**).

The perianth of a mature flower consisted of two whorls: a green-red, thick calyx, fused at the base and typically pentamerous (visible from St2), and an urceolate, deciduous, sympetalous corolla with five short lobes ranging from recurved to revolute (**Figure 1b**, St7). At St1, bulges appeared at the inflorescence apex, protected by an extrafloral bract with digitiform hairs (**Figures 1c, d**). The vegetative meristem transformed into generative tissue, with the tip becoming sporogenous and the base forming the receptacle meristem. As primordia grew outward, internal protuberances emerged (**Figures 1d, e**), elongating and curving into stamen primordia. At this stage, filaments and anthers were not yet distinguishable. Stamens formed first, surrounded by developing sepals and petals, followed by the pistil primordium centrally. Stamen development proceeded faster than that of the gynoecium and ovules (**Figures 1d, e**).

The calyx had a single epidermal layer with elongated cells on the adaxial side and rounded cells on the abaxial side. The mesophyll comprised several undifferentiated parenchyma layers without visible vascularization. Abaxial parenchyma cells were small and elongated, while adaxial cells were larger and cubic (**Figures 1d, e**). Calcium oxalate crystals were present on the inner sepal surface, likely providing structural support (**Figure 1e**).

The corolla consisted of adaxial and abaxial epidermis with small cells and few stomata. Its inner epidermis was densely covered with trichomes throughout development, likely aiding self-pollination by trapping pollen near the corolla opening (Sealy and Webb, 1950). The petal mesophyll had several layers of uniform parenchyma cells with wide lumens, and vascular bundles were regularly arranged and visible in cross-section (**Figure 1f**).

The androecium consisted of 10 stamens surrounding the gynoecium. Stamen filaments were densely covered with white hairs and showed a double curvature that disappeared by St6-7. Filament length was minimal at St2–3, reaching maximum extension at St7 (**Figure 1g**). Filaments had an outer uniseriate layer of cuboid cells and inner parenchyma with a single vascular bundle (**Figure 1f**). Between St5 and St6, anthers inverted from extrorse to introrse position, developing two tapered spurs at their apex, consistent with previous reports (Simpson, 2010; Martins et al., 2021) (**Figures 1g, h**). Most Ericaceae have poricidal anthers adapted for buzz pollination (Mesquita‐Neto et al., 2018; Kriebel et al., 2023). The reddish anthers varied in size (five large alternating with five small), each with four pollen sacs arranged in pairs, forming a butterfly-shaped cross section (**Figure 1i**). Anthers were subbasifixed, with connective tissue separating the two lobes, containing parenchyma and a single vascular bundle (**Figure 1i**). The archesporium, made of uni- or binucleate polygonal cells, differentiated between St1 and St2 (**Figures 1j, k**). Pollen formation began early and was mostly complete by St3 (**Figure 1i**). Microsporangia cross sections showed three layers: an epidermis (or exothecium, the outermost single-layered and protective portion formed by papillose cells), an endothecium (composed by elongated cells, sometimes lignified), and the tapetum (the innermost layer) (**Figures 1i, k**). Druses were detected between adjacent pollen sacs (**Figure 1i**). From St3 onward, tapetum cell walls degenerated to produce sporopollenin. The anthers dehisced through terminal pores, releasing pollen, typically in tetrads (40–65 µm), shortly after anthesis (**Figures 1g, i**). Each anther contains approximately 500 pollen units. As the morphology of individual pollen grains has already been described by Martins et al. (2021), we simply confirmed their findings in our samples. The urceolate flower structure facilitates contact between pollinators and anther spurs, triggering pollen release through the pores onto the insects’ bodies. Additionally, the downward-facing orientation of the flowers likely protects pollen from rain, enhancing its preservation (Bynum and Smith, 2001; Lin and Forrest, 2019).

**Figure 2:**
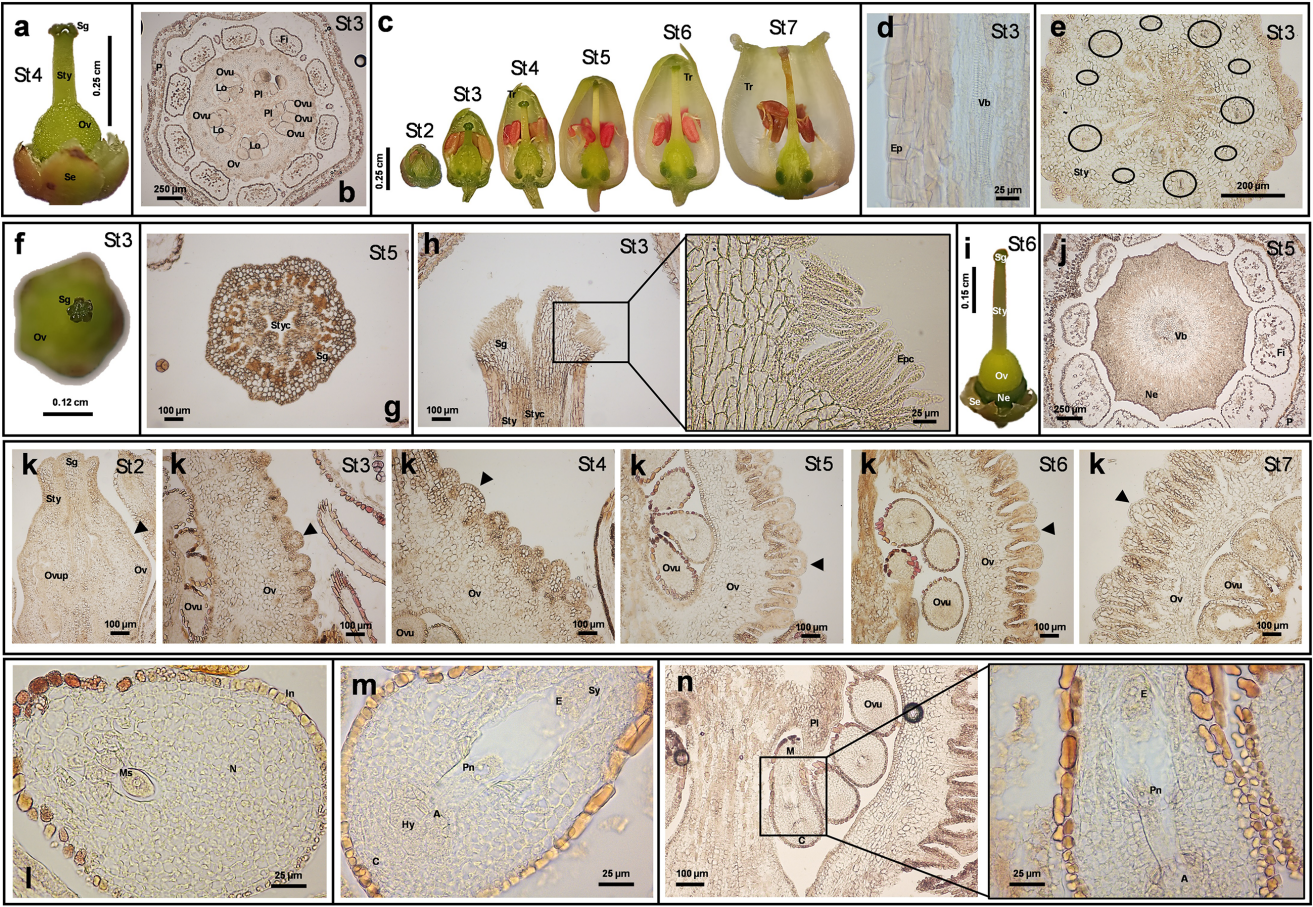
Anatomical images and histological sections from *Arbutus unedo* flower. The representative images reported in the figure were obtained by stereomicroscope (panels **a, c, f**, **i**) or optic microscope (panels b, d-e, g-h, and j-n). The dimension bar is reported for each panel. **(a)** Papillate pistil at St4. **(b)** Transversal section of flower at St3, showing the pentalocular ovary. **(c)** Style length and flower general morphology at different developmental stages. **(d)** Longitudinal section of the style at St3 showing the epidermal layer and some xylem lignified cells from the vascular bundle. **(e)** Transversal section of the style showing 10 collateral vascular bundles (included in black circles) and a central collenchymal area. **(f)** Image which visualizes the stigma lobes at St3. **(g)** Cross-section of the stigma at St5 showing the stylar canal. **(h)** Longitudinal section of stigma at St3 and relative magnification showing details of the stigmatic surface. **(I)** Image of the whole pistil, including the ring-shaped nectary. **(j)** Cross section at St5 showing stamen filaments compressed to the nectary. **(k)** Development of the ovary from St2 to St7: changes in shape and dimension of the protrusions (indicated by black arrows) characterizing the external portion of the ovary. **(l)** Immature ovule containing the megasporocyte. **(m, n)** Mature ovule containing the embryo sac (for panel n a magnification is also shown). (Legend: A, antipodal cells; C, chalaza; E, egg cell; Ep: epidermis; Epc, epidermal papillary cells; Fi, filament; Hy, hypostase; In, integument; Lo, locule; M, micropyle pole; Ms: megasporocyte; N, nucellus; Ne, nectary; Ov, ovary; Ovu, ovule; Ovup, ovule primordium; P, Petal; Pl, placenta; Pn, polar nuclei; Se, Sepal; Sg, stigma; Sty, style; Styc, stylar canal; Sy, synergids; Tr, trichome; Vb, vascular bundle).

The gynoecium appeared syncarpous, consisting of a single papillate pistil (**Figure 2a**). Initially, five distinct carpels developed and later fused into a superior, pentalocular ovary (**Figures 2b, c**). The ovary’s smooth outer wall became longitudinally furrowed from the style base to the receptacle (**Figure 2a**). The long, straight style, nearly as long as the petals (**Figure 2c**), featured a central canal (**Figure 2d**) and was composed of a monostratified epidermis, underlying parenchyma with 10 collateral vascular bundles, and central collenchyma (**Figures 2d, e**). From stage St2 onward, the pistil, style, and stigma were clearly distinguishable (**Figure 2c**). The stigma, located at the style apex, matched the number of carpels and ovary locules (**Figures 2f, g**), and was covered with elongated papillary cells (**Figure 2h**). A prominent intrastaminal nectary ring encircled the ovary base and receptacle (**Figure 2i**), with nectary slits extending toward the adjacent stamen filaments (**Figure 2j**). SEM images of these structures in *A. unedo* are shown in Erbar (2014). Microscopically, the ovary wall began as an epidermal layer over parenchyma, later developing protrusions due to outer parenchyma cell divisions, likely for storage. Our analysis revealed a thick cuticle on the epidermis, which progressively thickened as the ovary developed (**Figure 2k**). The five locules contained numerous axile ovules, which were unitegmic, anatropous, and crassinucellate (**Figures 2k–n**; see also Simpson, 2010). Ovules appeared as rounded bulges on the placentae (**Figure 2k**), which lacked visible vascular tissue, suggesting symplastic nutrient transport. Until stage St2, ovules were undifferentiated; by then, a megasporocyte became visible (**Figure 2l**). Ovule development occurred asynchronously from St3 to St6, even within the same locule. Despite difficulty in observing all stages of megasporogenesis and megagametogenesis, mature embryo sacs were 8-nucleate, 7-celled, and elongated, consistent with the *Polygonum* type, as reported in other Ericaceae (Palser et al., 1990; Melia et al., 2012). Once mature, ovules with fully developed female gametophytes (**Figures 2m, n**) entered apparent stillness asting 2–5 months, awaiting fertilization. This delay may contribute to the characteristically slow floral development of *A. unedo*, as pollen matures earlier, by stages St3-St4. The differential maturation of *A. unedo* reproductive structures cannot be attributed to dichogamy (e.g., protandry), as both anthers and corolla remain sealed until stage St7. This suggests alternative explanations, potentially involving specific microRNAs, hormonal gradients, or environmental cues. It is worth noting that a comparable delay occurs during fruit ripening. Interestingly, after fertilization, the zygote enters dormancy for about six months before its first division. Embryo development then proceeds slowly, taking several additional months to complete (Villa, 1982). This delay may also be influenced by molecular signals from the embryo sac. To complete this overview, **Table 1** reports the main morphometric data of floral structures across developmental stages.

### Phenolic metabolites tend to decrease during flower differentiation, except carotenoids and tannins

Flowers are among the most complex plant structures (Alvarez-Buylla et al., 2010), developing through tightly regulated stages of initiation, differentiation, cell division, expansion, and senescence. Hence, the complexity of flower bud production, growth and opening would reflect the overlapping of the different biological mechanisms involved in the various maturation stages (Kumar et al., 2008). Molecular events occurring during this progression may affect, or be affected by, the content and profile of plant secondary metabolites, an aspect investigated here in *A. unedo*.

**Figure 3:**
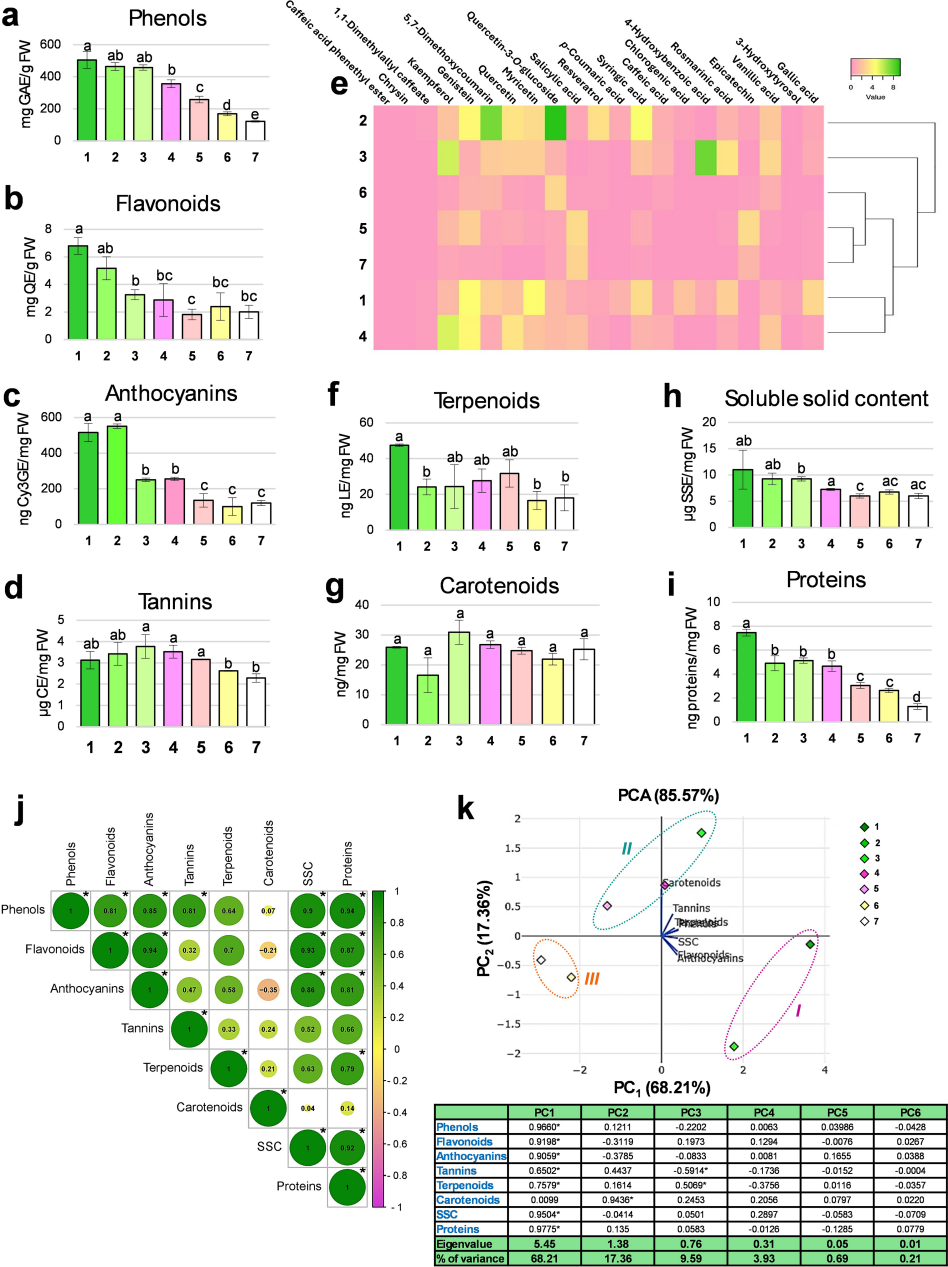
Biochemical analyses of *Arbutus unedo* flower. Histograms showing the total content in phenols **(a)**, flavonoids **(b)**, anthocyanins **(c)**, tannins **(d)**, terpenoids **(f)**, carotenoids **(g)**, soluble solid content **(h)** and proteins **(i)** measured by spectrophotometric assays at each flower stage (from 1 to 7) are reported. For each graph, the unit of measure is indicated on the *y*-axis. Results are reported as mean values ± standard error. Different letters indicate significant changes among the samples (*p*<0.05). Metabolomic data obtained by HPLC-DAD are shown in the form of a heatmap **(e)**, where analytes are reported in column and flower stages in row. Minimum and maximum values are set as pink and green, respectively. The hierarchical clustering based on the biochemical profiles of the samples is also visualized. Results of Pearson’s correlation analysis carried out among the investigated biochemical variables **(j)**: the correlation value is indicated inside each circle and asterisks indicate statistically significant correlations (*p*<0.05). Scatter plot derived from Principal Component Analysis (PCA) based on the studied biochemical traits; three clusters are detectable (*I*, early stages; *II*, intermediate stages; *III*, late stages). Correlation coefficients measured for the different variables (loadings) in each analysis (principal component, PC) are shown in the table reported below the graph, together with the respective Eigenvalue and % of variance (asterisks indicate significant values, *p*<0.05) **(k)**.

Total phenolic content decreased progressively, with modest reduction up to St3 and a sharp decline from St4 to St7. The highest phenolic concentration occurred at St1 (504.5 mg GAE/g FW), dropping to its lowest at St7 (121.65 mg GAE/g FW) (**Figure 3a**), in agreement with previous reports of a general decline in phenolics during floral maturation (Liu et al., 2015; Park et al., 2021; Önder et al., 2022). Phenolics are known to support antioxidant defense mechanisms (e.g., peroxidase/ascorbate system), delaying tissue senescence (Ahmad and Tahir, 2017); thus, the depletion observed at St7 likely reflects oxidative processes accompanying corolla expansion, pollination, and onset of aging.

Total flavonoid content followed a similar decreasing trend to phenolics, with significant reductions at St3 and St5. The highest concentration was recorded at St1 (6.8 mg QE/g FW), dropping to a minimum at St5 (1.82 mg QE/g FW) (**Figure 3b**). Flavonoids are involved in various physiological processes, including growth, flower pigmentation, and stress responses (Dias et al., 2021; Shen et al., 2022), and belong to the broader class of phenolic compounds. Their decline during flowering may reflect a metabolic shift, as flavonoid and lignin biosynthesis compete for the same precursor, *p*-coumaroyl CoA, at an early branch point in the phenylpropanoid pathway (Besseau et al., 2007). Despite their fragile appearance, *A. unedo* flowers may form thin lignified layers, particularly in the endothecium, potentially supporting reproductive success while limiting flavonoid accumulation (Borghi and Takeda-Kimura, 2024).

Among flavonoids, anthocyanins decreased markedly during development, especially at St3 and St5, mirroring the overall flavonoid trend. The highest concentration was found at St2 (551.06 ng Cy3GE/mg FW), and the lowest at St6 (100.19 ng Cy3GE/mg FW) (**Figure 3c**). Microscopy confirmed pigment accumulation in epidermal cells at St1 (**Figures 1a, c–e**), supporting a photoprotective role during early differentiation (Liu et al., 2018). Anthocyanins are present in most Angiosperms not only to provide different staining to petals during flower development (or to other plant elements) but also to prevent damages from ultraviolet light, pests, and diseases (Liu et al., 2018). From a biochemical perspective, some phenolic compounds, such as flavanones and dihydroflavonols (that are colorless and unstable compounds), can be oxidized and glycosylated to anthocyanins, becoming rather stable, colored and soluble in vacuoles. Their later decline coincides with conversion into flavanols and proanthocyanidins, present as both monomers (e.g., catechin) and oligomers (e.g., condensed tannins) (Bogs et al., 2005) and is consistent with the white corolla of mature flowers (Wang et al., 2023). The possibility that colorless flavonoids (e.g., flavones, flavonols) act as UV guides for pollinators, as in other white-flowered species, remains open (Ahmad et al., 2023).

Tannin content remained relatively stable, from 3.77 µg CE/mg FW at St3 to 2.63 µg CE/mg FW at St6, with a slight but significant decline between St5 and St6 (**Figure 3d**). This reduction coincides with the completion of embryo sac formation. While earlier studies have detected tannins only in ovary tissues (Villa, 1983), our results show their persistence across floral development. Their distribution likely reflects both developmental roles, such as masking polysaccharides and proteins during ovule differentiation (Ma et al., 2019), and defensive functions against biotic stress, in line with their protective role described decades ago (Nitao and Zangerl, 1987). The presence of tannins throughout the entire developmental process likely reflects a constant protective demand, particularly for the carpels and stamens during the early phases, against possible external damage. Indeed, it is known that in mature flowers proanthocyanidins gradually diminish (**Figure 3d**) (Robil and Tolentino, 2015).

Given the overall decline in phenolic compounds across developmental stages, we profiled 21 specific aromatic secondary metabolites by HPLC-DAD (**Supporting Information 2**). The heatmap (**Figure 3e**) revealed the greatest phytochemical variation in early buds (St1-St4), while St5-St7 clustered together due to their uniformly low phenolic levels, confirming the decreasing trend observed with spectrophotometric assays. Early-stage buds were characterized by notable accumulation of phenolic acids (e.g., vanillic, rosmarinic, 4-hydroxybenzoic, syringic acids), 5,7-dimethoxycoumarin, and flavonoids such as resveratrol, myricetin, and quercetin (Schmitzer et al., 2009, 2013). The molecules reaching the highest levels among others were quercetin-3-*O*-glucoside (9.29 mg SE/g FW) and 5,7-dimethoxycoumarin (7.4 mg SE/g FW) in St2, 4-hydroxybenzoic acid (7.73 mg SE/g FW) in St3, and kaempferol both in St3 (5.69 mg SE/g FW) and St4 (5.38 mg SE/g FW). The content of syringic acid, myricetin, genistein and quercetin, which touched their maximum values in the early stages (i.e., respectively, 4.67 mg SE/g FW in St2, 4.48 mg SE/g FW in St1, 4.62 mg SE/g FW in St1, and 3.41 mg SE/g FW in St2), tended to decrease during the development, becoming negligible at St7. Some substances were detected in concentration higher than 2 mg/g FW only at single stages; examples are: gallic acid (2.78 mg SE/g FW at St1), resveratrol (3.17 mg SE/g FW at St2), rosmarinic acid (3.51 mg SE/g FW at St3), and epicatechin (2.98 mg SE/g FW at St5). Vanillic acid appeared in discrete amounts during the first three stages (St2: 2.16 mg SE/g FW; St3: 2.84 mg SE/g FW; St4: 2.16 mg SE/g FW), while salicylic acid (St5: 3.10 mg SE/g FW and St7: 2.67 mg SE/g FW) and epicatechin (St5: 2.98 mg SE/g FW and St7: 1.39 mg SE/g FW) in the final ones. Curiously, chrysin, 1,1-dimethylallyl caffeate and caffeic acid phenethyl ester did not seem to typify any developmental stage, remaining in traces in all samples. The functional interpretation of these profiles remains complex, as many phenolics share overlapping antioxidant, protective, and regulatory roles (Landi et al., 2015; Davies et al., 2018). In this scenario, some hypothetical functions of these compounds could be postulated. The low levels of certain phytochemicals, such as caffeic acid and its derivatives, in late developmental stages (St4-St7) may reflect ecological selection, as pollinators prefer nectar with reduced concentrations of these compounds (Hagler and Buchmann, 1993). In contrast, salicylic acid, a hormone in plant immunity and stress responses, including transcriptional reprogramming (Ding and Ding, 2020), increased only in mature stages, possibly as a preparatory signal for anthesis and potential biotic stress. High levels of phenolics, particularly flavonoids, in early stages likely reflect their protective roles against UV radiation and heat stress during summer–early autumn flowering (Ferreyra et al., 2021). Additionally, flavonoids (e.g., myricetin) act as potent antioxidants and regulators of redox homeostasis, modulating gene expression and mitigating oxidative stress from photooxidation or chloroplast-derived reactive oxygen species (Xie et al., 2013; Mekawy et al., 2018; Muñoz and Munné-Bosch, 2018), which may explain their dynamic content across developmental stages.

Chemical analysis was extended to total terpenoids and carotenoids. Terpenoid levels remained consistently low throughout development, with a slight decline from St2, ranging from 47.5 ng LE/mg FW at St1 to 16.57 ng LE/mg FW at St6 (**Figure 3f**), suggesting a limited role in *A. unedo* despite their known contribution to floral scent (Mileva et al., 2021). On the other hand, total carotenoids were relatively stable, varying from 30.93 ng/mg FW at St3 to 16.53 ng/mg FW at St2 (**Figure 3g**). Carotenoids, derived from the terpene pathway, are typically involved in tissue pigmentation. However, given the white coloration of *A. unedo* flowers, their constant levels suggest alternative functions such as light harvesting, photoprotection, and serving as precursors for abscisic acid biosynthesis, roles likely crucial during flower development (Hirschberg, 1999).

The concentration of soluble solids, primarily sugars, ranged from 11 µg SSE/mg FW in St1 to 6 µg SSE/mg FW in St5, with significant decrease in the timeframe St3-St5 (**Figure 3h**). This reduction trend in sugar content was consistent with observation in other species (Gul et al., 2015; Park et al., 2021). Soluble carbohydrates serve multiple roles-as respiratory substrates, structural components, metabolic precursors, regulators of petal pigmentation, and contributors to osmotic balance, thereby facilitating flower growth and petal expansion (Bieleski, 1993; Moalem-Beno et al., 1997; Norikoshi et al., 2016). The high content at St1 likely supports the respiratory demands of the sporogenous meristem, while in later stages, soluble solids may support cell expansion by loosening membrane integrity and promoting the synthesis of colorless metabolites.

Lastly, total protein content followed a similar declining trend: the highest content was detected in the youngest stage (St1: 7.47 ng/mg FW), whereas the lowest one in St7 (1.28 ng proteins/mg FW) (**Figure 3i**). Significant decreases were observed at St2, St5 and St7, as already reported in the literature (Sood et al., 2006).

A correlation analysis (**Figure 3j**) revealed significant positive relationships among most biochemical parameters. Phenols correlated positively (magnitude between 0.81 and 0.94) and significantly with all the other classes of metabolites, except terpenoids and carotenoids, and with sugars and proteins. Flavonoids were closely aligned with anthocyanins (0.94) but also correlated with sugars (0.93) and proteins (0.87). Anthocyanins, like the previous phytochemicals, significantly correlated with sugars (0.86) and proteins (0.81), whereas tannins only with total phenols (0.81). This is explained by phenolics being precursors of complex compounds like lignin and tannins, all deriving from the same biosynthetic pathway (Cheynier et al., 2013). Phenolics are often glycosylated to enhance vacuolar solubility and membrane transport, sometimes at ratios exceeding one-to-one (Šamec et al., 2021). Our spectrophotometric data show that the gradual sugar decline from St1 to St7 parallels the reduced energy demand for secondary metabolism, reflected by the concurrent decrease in phenolics. By contrast, carotenoids did not correlate significantly with any group of substances, and even negatively with flavonoids (-0.21) and anthocyanins (-0.35), whereas terpenoids appeared to be in line with proteins (0.79).

To investigate the existence of possible relationships among the seven developmental stages of *A. unedo* flower, a Principal Component Analysis (PCA) was carried out, providing as input the results of previous analyses (**Figure 3k**). This statistical method produced a plot based on the only 2 functions (out of 6, that is PC_1_ and PC_2_) showing an Eigenvalue greater than 1 and able to encompass together the 85.57% of the total observed variance. PC_1_, alone, explained the 68.21% of total variability measured in the samples. Carotenoids showed no significant correlation with this component, but they were the only biochemical category that positively contributed to the second factor (PC2), which represented the 17.36% of the variance. In the scatter plot, 3 clusters could be traced. In detail, the group *I* included the early developmental stages (i.e., St1 and St2) with the highest values of flavonoids and anthocyanins. In the cluster *II*, the intermediate growth stages (i.e., St3, St4 and St5) were grouped; they were placed on the positive side of PC_2_, showing important levels of carotenoids and tannins. The late stages (St6 and St7), instead, could be found in the cluster *III*, located on both negative sides of the graph. All samples fell within the 95% confidence ellipse. In conclusion, flavonoids likely play a key protective role in early bud development, while metabolites like carotenoids appear to act later, possibly conferring resistance during the extended dormancy up to St5. The stages before anthesis, instead, are marked by a general decline in metabolite levels.

The final step of chemical characterization consisted in a GC-MS analysis, to investigate the lipophilic fraction of the floral stages. We detected 7 straight-chain saturated fatty acids (i.e., hexadecanoic acid, heptacosanoic acid, octadecanoic acid, docosanoic acid, eicosanoic acid, docosanoic acid, and tetracosanoic acid), 2 omega-6 (i.e., 9,12-octadecadienoic acid and 9,12,15-octadecatrienoic acid) and 1 omega-3 (i.e., 11,14,17-eicosatrienoic acid) polyunsaturated fatty acids, one phytosterol (i.e., beta-sitosterol), one fatty alcohol (i.e., lignoceric alcohol) and the vitamin E (**Supporting Information 2**). These hydrophobic compounds are common components of cell membranes, plant oils and waxes, with vitamin E contributing antioxidant protection. GC-MS profiles were largely consistent across stages, except for some analytes (i.e., eicosanoic acid, tetracosanoic acid and lignoceric alcohol), which appeared uniquely in St4 or St5. This may reflect androecium maturation, including cuticle deposition, lignified cell differentiation, and pollen wall reinforcement in the anthers (Piffanelli et al., 1997; Zhang et al., 2016). Hexadecenoic acid (palmitoleic acid) and 9,12-octadecadienoic acid (linoleic acid) resulted the only molecules present at all stages. Although data on floral fatty acid composition are limited, high linoleic acid levels in petals, ovary, and anthers before anthesis reported by Matsuzaki et al. (1983) align with our observations.

### Flavanol biochemical pathway resulted unexpectedly active at alternate stages

**Figure 4:**
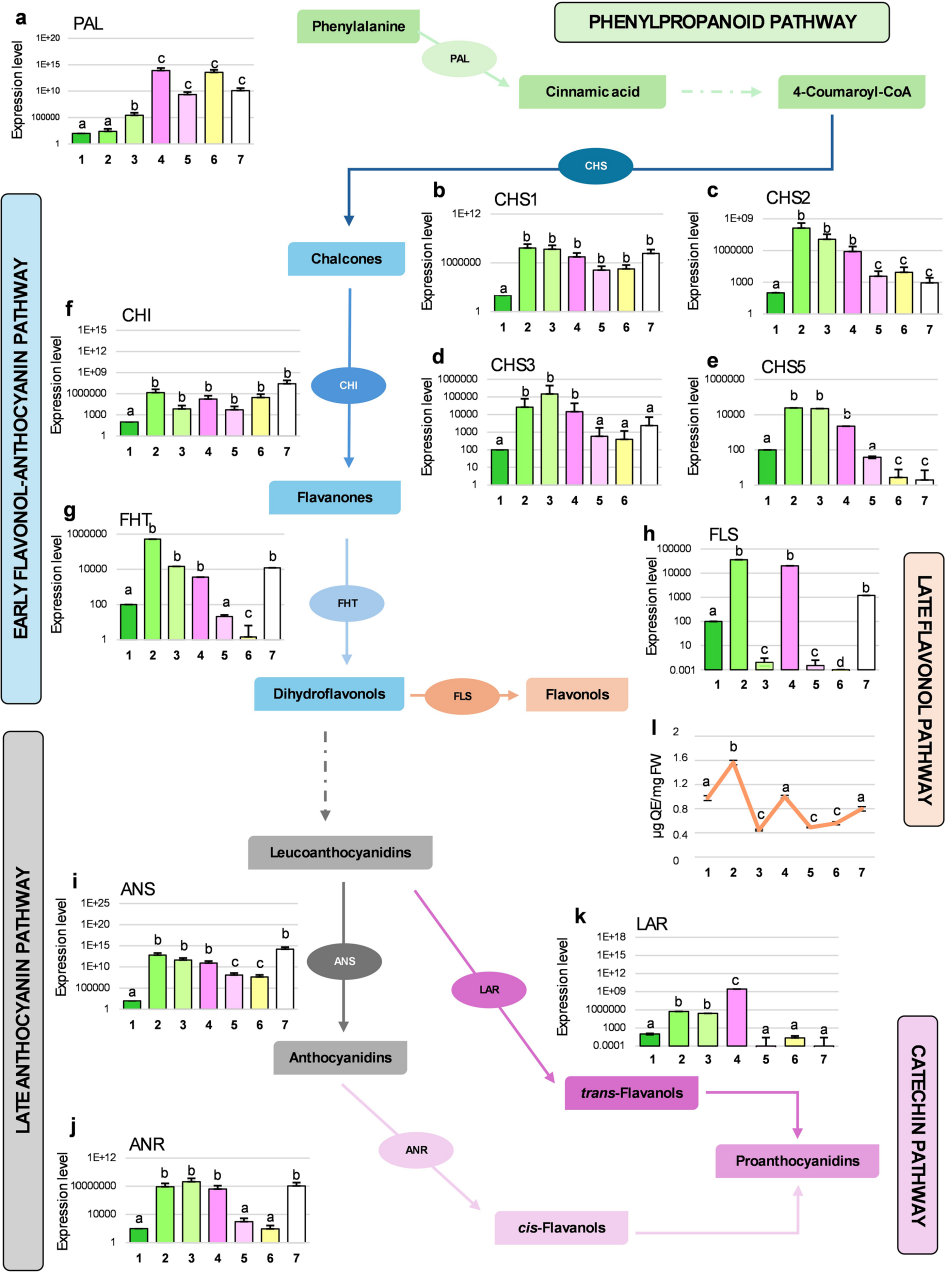
qPCR analyses of the expression levels for 11 genes involved in the biosynthetic pathway of phenylpropanoids and flavonoids (schematically drawn by reporting enzymes and relative products; dotted arrows indicate the presence of intermediate steps not shown) in *A. unedo* flower samples (from stage 1 to 7). mRNA levels in the various samples were normalized with respect to β-*Actin* transcript content and compared to the Stage 1 (considered as unit, 100). Results are shown as mean values ± standard error and different letters indicate significant changes among the samples (*p*<0.05). In panel **l**, the total content of flavonols is reported (here the unit of measure is indicated on the *y*-axis). Legend: *PAL*, Phenylalanine ammonia-Lyase **(a)**; *CHS1*, Chalcone synthase 1 **(b)**; *CHS2*, Chalcone synthase 2 **(c)**; *CHS3*, Chalcone synthase 3 **(d)**; *CHS5*, Chalcone synthase 5 **(e)**; *CHI*, Chalcone isomerase **(f)**; *FHT*, Flavanone 3-β-hydroxylase **(g)**; *FLS*, Flavonol synthase **(h)**; ANS, Anthocyanidin synthase **(i)**; ANR, Anthocyanidin reductase **(j)**; *LAR*, Leucoanthocyanidin reductase **(k)**.

Biochemical analyses indicated that phenolic compounds were the most dynamically modulated secondary metabolites during flower bud development. To assess potential regulation at the gene expression level, qPCR analysis was performed on 11 genes involved in flavonoid biosynthesis across the developmental stages of *A. unedo* (**Figure 4**).

PAL mRNA constantly increased during the flower development (**Figure 4a**), particularly from St4 onwards. While PAL is the first key enzyme in the synthesis of phenolics and aromatic amino acids, our spectrophotometric and chromatographic analyses showed a decline in total phenols, flavonoids, and proteins between St3 and St7. This apparent discrepancy highlights a common phenomenon, where transcript abundance does not always reflect the respective enzyme activity and, consequently, the level of phytochemical product. One plausible explanation could be that, in the last stages of development, PAL activity is redirected toward monolignol production for lignin biosynthesis, supporting structural reinforcement in mature floral organs such as anthers (Yadav, 2018), and contributing to sporopollenin enrichment in lignin subunits (guaiacyl, *p*-hydroxyphenyl, and syringyl) (Yang et al., 2023). However, the observed increase in PAL transcript levels during late flower stages may result from feedback triggered by phenolic depletion, without a corresponding rise in its protein synthesis or stability. This observation underscores the complex regulation of the phenylpropanoid and flavonoid pathways in plants and the frequent divergence between transcriptomic and metabolomic profiles (Lepiniec et al., 2006; Yoon et al., 2015). Early flavonoid biosynthesis, beginning with chalcone formation, involves CHS and CHI (**Figures 4b–f**). Four CHS isoforms (CHS1–3, CHS5) displayed a peak at St2 followed by a gradual decline, whereas CHI remained relatively constant after the initial St2 accumulation (**Figure 4b–f**). FHT, converting flavanones in dihydroflavanols, mirrored CHS expression but exhibited a pronounced increase at St7 (**Figure 4g**). The expression of FLS (**Figure 4h**) was quite in line with the data obtained by liquid chromatography, reflecting accumulation of quercetin, quercetin-3-*O*-glucoside and kaempferol at St2 and St4. Indeed, FLS displayed a distinct alternating activation at St2, St4, and St7, which was supported by a parallel accumulation of flavonols in the same stages (see next paragraph and **Figure 4l**), suggesting a possible stage-specific role in regulating phytohormones, cell growth, differentiation, and signaling (Brunetti et al., 2018). Overall, the maximum expression of CHS, FHT and FLS occurred in the immature buds (i.e., St2), supporting the evidence already documented in Stich et al. (1992). Late flavonoid biosynthetic genes, ANS, ANR and LAR showed stage-specific trends. ANS, which catalyzes the reaction from leucoanthocyanidin to anthocyanidins, presented the same trend of FHT (**Figure 4i**). The decrease in its mRNA levels towards St5 and St6 would reflect the results of the spectrophotometric assay for total anthocyanins; however, a re-accumulation of this transcript at St7 was found. ANR, which directly produces epicatechins, followed the same expression pattern of ANS (**Figure 4j**). The high level of ANR mRNA at St4 and St7 could justify the peaks of epicatechin detected by HPLC at St5 and St7. At St7, the up regulation for *FHT*, *FLS*, *ANS* and *ANR* genes and the simultaneous reduction of total flavonoids could seem contradictory at first glance; however, it might be explained by the fact that strawberry tree flowers are white. Indeed, petals with this type of coloration typically accumulate phenolic acids and flavan-3-ols (Ahmad et al., 2023). Lastly, LAR expression increased from St1 to St4, then dropped significantly in the final three stages (**Figure 4k**), reflecting the reduction in tannin content observed in the spectrophotometric analysis. Similar declines in flavonoid enzyme expression during flower development were reported in *Brassica oleracea* var. *acephala* by Lan et al. (2017), who have linked this trend to increased reactive oxygen species (ROS), suggesting their involvement in stigma maturation, pollination, and fertilization. This mechanism may also explain the reduced levels of antioxidant phytochemicals observed in the late stages of *A. unedo* flower development, as revealed by our spectrophotometric and chromatographic analyses.

### Temporally and spatially regulated expression of flavonols suggests unconventional functions for these phytochemicals in *A. unedo* flower maturation

Taken together, the data indicate that flavonols are among the most dynamically modulated secondary metabolites during *A. unedo* flower development, both in content and gene expression. Ubiquitous in plants, flavonols contribute to UV protection, antioxidant defense, and responses to environmental stressors, but also participate in male fertility, pollen-stigma interactions, plant architecture regulation, and other molecular processes (Mo et al., 1992; Ylstra et al., 1992; Wang et al., 2024). Notably, flavonols can modulate actin function (Böhl et al., 2007). The coexistence of both canonical (e.g., antioxidant) and non-canonical (e.g., signaling mediator) roles highlights flavonols as a particularly promising class of phytochemicals for further study.

In fruit trees, flavonols also play key roles in floral regulation. In peach (*Prunus persica*), R2R3-MYB factors, such as PpMYB15 and PpMYBF1, directly regulate FLS expression, enhancing flavonol accumulation while reducing anthocyanins, thus affecting flower pigmentation (Ravaglia et al., 2013; Cao et al., 2018). Similarly, in apple (*Malus domestica*), MdMYB8 activates MdFLS, promoting flavonol biosynthesis independently of anthocyanin pathways, with levels further modulated by UV light (Henry-Kirk et al., 2018; Li et al., 2020). These examples highlight that stage- and tissue-specific flavonol regulation is at least partly a conserved feature across woody perennials, supporting our observation that *A. unedo* exhibits temporally and spatially restricted flavonol accumulation.

According to all this premise, we measured total flavonol content using a specific spectrophotometric assay (**Figure 4l**), revealing a trend closely matching the *FLS* gene expression profile obtained by qPCR (**Figure 4h**). The observed continuous synthesis and degradation of flavonols supports their involvement in temporally peculiar biological roles during *A. unedo* flower development. We therefore investigated their distribution across the different floral tissues. Flavonoids can be transported over long distances to reach target sites where their activity is required (Buer et al., 2007; 2008). Flavonols are naturally fluorescent, and their detection is enhanced by diphenylboric acid 2-amino ethyl ester (DPBA), a reagent that forms highly fluorescent complexes with these compounds. Notably, quercetin (Q)-DPBA and kaempferol (K)-DPBA complexes exhibit distinct emission spectra, enabling their *in-situ* localization (Vu et al., 2015; Nguyen, 2020).

**Figure 5:**
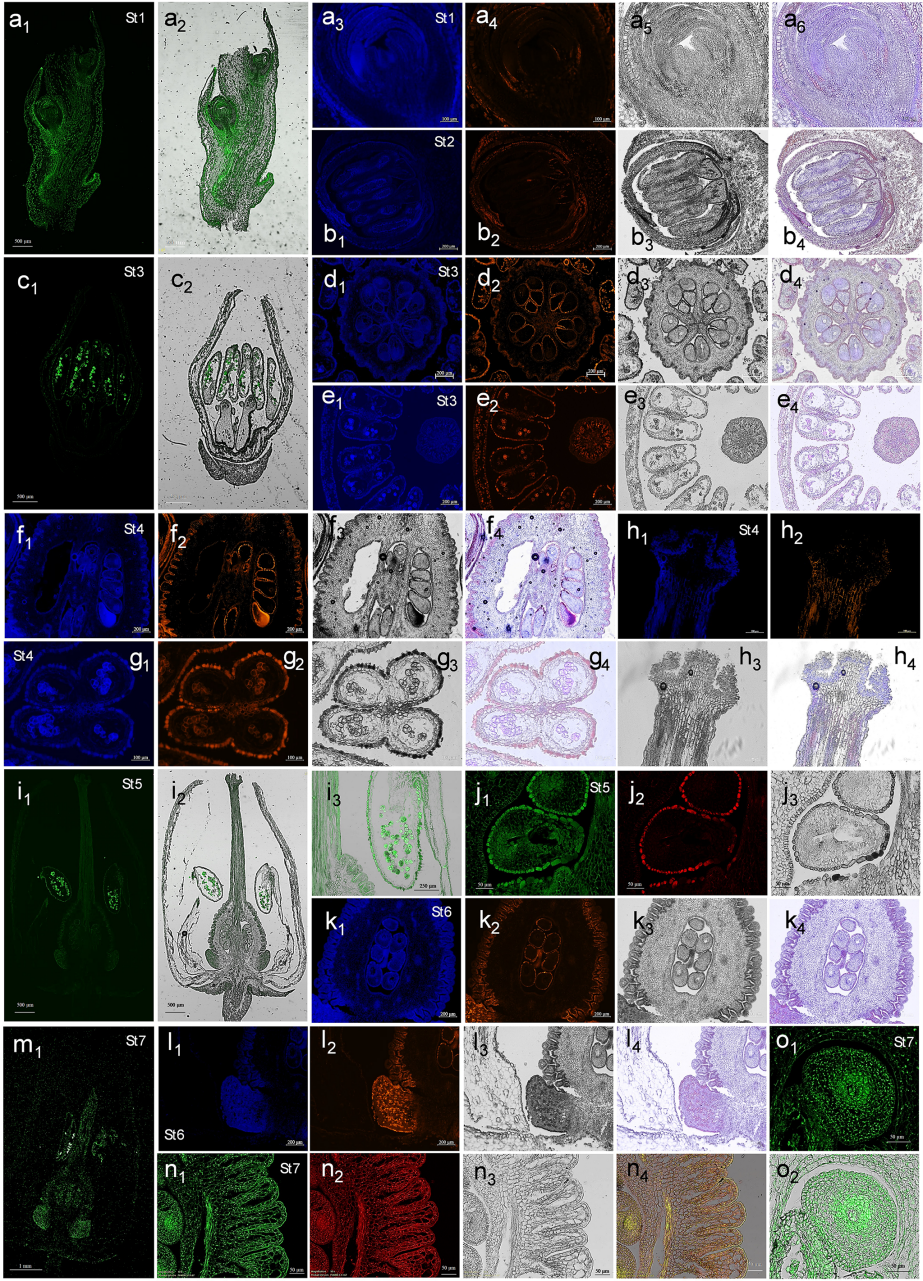
Histochemical localization of flavonols in *Arbutus unedo* samples. Representative images of histological sections at different developmental stages observed by fluorescence microscopy after DPBA staining. Kaempferol (K)-DPBA and quercetin (Q)-DPBA complexes were discriminated collecting fluorescence at different emission ranges, as reported in method section. (Series of panels **a**) *Samples from St1.* K-DPBA visualization in the longitudinal section of a flower bulge included into a bract **(a_1_)** and its merging with bright field **(a_2_)**. Detail of a flower bud in longitudinal section: **(a_3_)**, K-DPBA; **(a_4_)**, Q-DPBA; **(a_5_)**, bright field; and **(a_6_)**, merged channels. (Series of panels **b**) *Samples from St2*. Detail of a flower bud in transversal section: **(b_1_)**, K-DPBA; **(b_2_)**, Q-DPBA; **(b_3_)**, bright field; and **(b_4_)**, merged channels. (Series of panels **c, d** and **e**) *Samples from St3.* K-DPBA visualization in the longitudinal flower section **(c_1_)** and its merging with bright field **(c_2_)**. Transversal section of ovary: **(d_1_)**, K-DPBA; **(d_2_)**, Q-DPBA; **(d_3_)**, bright field; and **(d_4_)**, merged channels. Transversal section of anthers (series e): **(e_1_)**, K-DPBA; **(e_2_)**, Q-DPBA; **(e_3_)**, bright field; and **(e_4_)**, merged channels. (Series of panels **f, g** and **h**) *Samples from St4.* Longitudinal section of ovary (series f): **(f_1_)**, K-DPBA; **(f_2_)**, Q-DPBA; **(f_3_)**, bright field; and **(f_4_)**, merged channels. Cross section of a single anther (series g): **(g_1_)**, K-DPBA; **(g_2_)**, Q-DPBA; **(g_3_)**, bright field; and **(g_4_)**, merged channels. Longitudinal section of stigma (series h): **(h_1_)**, K-DPBA; **(h_2_)**, Q-DPBA; **(h_3_)**, bright field; and **(h_4_)**, merged channels. (Series of panels **i** and **j**) *Samples from St5.* K-DPBA visualization in the longitudinal section of a flower **(i_1_)** and its merging with bright field **(i_2_)**. A magnification of the right anther shown in **(i_2_)** panel is also reported **(i_3_)**. Transversal section of an ovule (series j): **(j_1_)**, K-DPBA; **(j_2_)**, Q-DPBA; and **(j_3_)**, bright field. (Series of panels **k** and **l**) *Samples from St6*. Longitudinal section of ovary (series k): **(k_1_)**, K-DPBA; **(k_2_)**, Q-DPBA; **(k_3_)**, bright field; and **(k_4_)**, merged channels. Longitudinal section of nectary (series l): **(l_1_)**, K-DPBA; **(l_2_)**, Q-DPBA; **(l_3_)**, bright field; and **(l_4_)**, merged channels. (Series of panels **m, n** and **o**) *Samples from St7.* K-DPBA visualization in the longitudinal section of a flower **(m_1_)**. Longitudinal section of ovary (series n): **(n_1_)**, K-DPBA; **(n_2_)**, Q-DPBA; **(n_3_)**, bright field; and **(n_4_)**, merged channels. K-DPBA visualization in the longitudinal section of ovule **(o_1_)** and its merging with bright field **(o_2_)**. The dimension bar is reported for each panel.

**Figure 5** shows representative images of kaempferol and quercetin spatial distribution of in the strawberry tree samples, after staining with DPBA. Fluorescence was consistently visible in the outer layer of anthers and pistil (i.e., stigma, style and ovary), as well as in pollen and the outer integument of ovules, from St2 to St7. By contrast, primordia of the flower buds (St1), observed in longitudinal section, displayed diffuse throughout the structure, with stronger signals observed in vascular tissues, internal surfaces of bracts and surfaces of petal primordia compared to the bulges (**Figures 5a_1_, a_2_**).

Microscopy analysis evidenced how the investigated flavonoids were mainly distributed in the cytoplasm of cells (**Figure 5**, series of panels a-l), suggesting short-range, symplastic movements or *in situ* synthesis. However, in anthers, style and ovary from St4 to St7 (for example see **Figure 5**, panels g_2_ and h_2_ and the series of n panels), flavonols could be clearly detected in intercellular spaces, highlighting long-distance apoplastic transport in these mature tissues. Notably, at St7, both these two phytochemicals were observed in the apoplast and in the nuclei of ovary and ovule cells (**Figure 5**, series of panels n and o). Previous studies have shown that nuclear-localized flavonols can be synthesized by specific nuclear isoforms of biosynthetic enzymes, suggesting potential interactions with DNA (Saslowsky et al., 2005; Feucht et al., 2008; Kuhn et al., 2011; Zhang et al., 2020). Therefore, the peak in *FLS* transcript-level and total flavonols biosynthesis registered only at St2, St4 and St7 (**Figures 4h, i**) may reflect the capacity of these secondary metabolites to regulate gene expression in a time and space specific manner. This hypothesis requires further confirmation to clarify the molecular mechanisms involved in *A. unedo* flower development.

Kaempferol displayed a broadly diffused and intense signal in early buds (**Figures 5a_3_, a_6_**), stamen primordia (**Figures 5b_1_, b_4_**) and immature female reproductive structures (**Figures 5d_1_, d_4_, f_1_, f_4_, k_1_, k_4_**), whereas quercetin was less prominent and mainly localized to their peripheral cell layers (**Figures 5a_4_, b_2_, d_2_, f_2,_, k_2_**). This result aligns with previous studies reporting that flowers predominantly contain kaempferol and its derivatives (Shirley et al., 1995; Burbulis et al., 1996; Routaboul et al., 2006).

Kaempferol accumulated notably in the androecium from St3 to St7, especially in pollen grains, which exhibited strong fluorescence (**Figure 5**, panels of the series c, g, and i and image m_1_). At St3 (**Figure 5**, series of panels e), this metabolite was also detected in tapetum cells, whose programmed degeneration released vesicles (i.e., tapetosomes) into the anther locule containing microspore coat precursors (**Figure 5**) (Stanley and Linskens, 1974; Wu and Cheung, 2000). As expected, considering that phenolics are important components of the sporopollenin (Xue et al., 2023), strawberry tree pollen resulted characterized also by the presence of quercetin, although to a lesser extent compared to the other flavonol (**Figures 5e_2_, g_2_**). Lastly, in the same images, it was clear that exothecium, made up of numerous papillose cells, contained high level of both quercetin and kaempferol, reflecting their roles in pollen germination, redox balance and coating (Mo et al., 1992; Ylstra et al., 1992; Lan et al., 2017; Wang et al., 2024).

From St1 to St6, ovule cells appeared rich in kaempferol but lacking for quercetin, whereas their integuments contained high levels of both flavonols (**Figure 5**, series of panels d, f and j). This phenomenon is clearly visible in the series of panels k in **Figure 5**. At St7, mature ovules displayed a notable shift: kaempferol and quercetin appeared in internal ovule tissues, while signals in the integuments decreased (**Figure 5**, series of panel n and o). Considering that the synthesis of kaempferol and quercetin tended to be constant between St6 and St7, according to HPLC-DAD data (**Figure 3e**), the most plausible explanation is their migration from integument cells to the female gametophyte cells. This would account for their apoplastic localization at St7. It seems unlikely these metabolites are degraded in the integuments and then resynthesized in the ovule, as this would require high energy expenditure. Cytoplasmic accumulation was observed exclusively in the central ovule cells (**Figure 5**, series of panel n and o), likely reflecting a role in mitigating ROS-induced damage during female gametogenesis and fertilization, as oxidative environments are required for pollen tube reception (Martin et al., 2013). Alternatively, the distribution of quercetin may relate to auxin regulation during megagametophyte maturation. Auxin maintains placental meristematic activity, megasporangium identity, and induces ovule initiation, somatic-to-germline transition, and megasporogenesis (Benková et al., 2003; Ceccato et al., 2013; Cucinotta et al., 2014; Reyes-Olalde and de Folter, 2019). Therefore, while auxin’s functions in ovules before and after fertilization are well established, detailed information on its precise localization during specific developmental time points remains limited (Kuusk et al., 2002; Benková et al., 2003; Larsson et al., 2017). Quercetin, which binds auxin transporters more strongly than kaempferol (Lewis et al., 2011; Luo et al., 2016), may inhibit auxin activity in ovule outer layers from St1 to St6, allowing hormone action in central regions to promote megaspore formation and egg cell development. At St7, quercetin likely acts as an endogenous negative regulator of auxin transport in both sporophytic and gametophytic tissues.

Analysis of kaempferol localization in longitudinal flowers sections at St5 (**Figure 5**, panels i_1_ and i_2_) revealed general staining in nectary glands at the basis of the pistil. At St7, DPBA signal intensified in these structures (**Figure 5**m_1_), indicating kaempferol accumulation. This is consistent with the role of flavonoids in nectar, where they can attract specialist pollinators, deter undesired visitors, alter insects’ behavior, provide antimicrobial protection, and contribute to the nectar redox balance (Gismondi et al., 2024). However, at St6, while kaempferol appeared quite homogenously diffused in the tissue, quercetin unexpectedly accumulated in the entire epidermal monolayer of the nectary and only in some of its parenchyma cells (**Figure 5**, series of panels l). The same fate seemed to occur for kaempferol at St7 (**Figure 5m_1_**). To our knowledge, this specific quercetin distribution in nectaries has not been previously reported; it is possible that quercetin, together with naringenin, acts as a minor nectar component that may deter pollinators by reducing nectar palatability (Hernández et al., 2019). Finally, as noted, the two flavonols were widely distributed in both the apoplast and nuclei of ovule and ovary tissues at St7. Only the central ovule cells retained flavonols in the cytoplasm, possibly reflecting their auxin-related roles in preparing for fertilization and supporting seed and fruit development.

## Conclusions

Flowering is essential for plant ecological, reproductive, and evolutionary success. In *A. unedo*, the complete flower production is unusually prolonged, suggesting molecular mechanisms that delay maturation. This study provides morphological and molecular insights into flower developmental stages, revealing a slower embryo sac maturation compared to male tissues, likely regulated by microRNAs, hormones, and environmental factors. We found that secondary metabolites reduced significantly during the growth, while flavonols accumulate at specific ontogenetic stages, as confirmed by the increased expression of their biosynthetic genes. Fluorescence microscopy showed flavonols initially in the cytoplasm of young tissues, later moving to intercellular spaces and nuclei, suggesting roles in signaling and gene regulation. These results highlight flavonols as key elements in *A. unedo* flower development, acting beyond their conventional roles by influencing hormones, cytoskeleton, and transduction pathways. Overall, this work advances understanding of *A. unedo* flowering, emphasizing flavonols’ central role in this process. Future studies should focus on their molecular functions and interactions with hormonal and environmental signals to clarify this reproductive adaptation mechanism.

## Data Availability

The original contributions presented in the study are included in the article/**Supplementary Material**. Further inquiries can be directed to the corresponding author.

## Supplementary material

Supplementary Table 1 Primers used in qPCR analyses.

Supplementary Table 2 Raw data from HPLC-DAD and GC-MS analyses. The concentration of secondary metabolites detected in *A. unedo* flower samples by HPLC-DAD analysis is reported. Data are expressed as mg of standard equivalent (mg SE) per g of plant fresh weight (g FW) and reported as mean values ± standard error. For GC-MS analysis, the list of molecules detected in each floral stage is reported. Results are reported as relative abundance.
