# Supplementary material for: Megagametophyte maturation dynamics and flavonol changes during Arbutus unedo flower development
Source: Front Plant Sci. 2025 Nov 6;16:1694629. doi: 10.3389/fpls.2025.1694629 (PMC12631325; doi:10.3389/fpls.2025.1694629)
Supplement: Supplementary Table 1 — Primers used in qPCR analyses. [file Table1.docx]

**SUPPORTING INFORMATION 1**

**SI1 - Primers used in qPCR analyses.**

| **Name of genes** | **Oligo name** | **5’-3’ Sequence** |
| --- | --- | --- |
| ***Anthocyanidin reductase*** | ANR F | CCTGAATACAAAGTCCCGACTGAG |
|  | ANR R | GTACTTGAAAGTGAACCCCTCCTTC |
| ***Anthocyanidin synthase*** | ANS F | GACTTGTCCATTTGGCCTC |
|  | ANS R | CCCCCTCAGTTCCTTAGCATACTC |
| ***Chalcone isomerase*** | CHI F | GCCGGAAATGGGAAAGTG |
|  | CHI R | GCTCAGTTTCATGCCTTGAC |
| ***Chalcone synthase*** | CHS1 F | GGCTCACCGTCGAGACCG |
|  | CHS1 R | GGAGAAGATCACTCGAATCA |
| ***Chalcone synthase*** | CHS2 F | GGCTCACCGTCGAGACCG |
|  | CHS2 R | GGTGAACCCAGATACCTTC |
| ***Chalcone synthase*** | CHS3 F | GGCTCACCGTCGAGACCG |
|  | CHS3 R | TCGAAACCCGGATGCCA |
| ***Chalcone synthase*** | CHS5 F | GGCTCACCGTCGAGACCG |
|  | CHS5 R | AGTGAACCCAGATACCTTT |
| ***Flavanone 3-β-hydroxylase*** | FHT F | ATCACCGTTCAACCTGTGGAAG |
|  | FHT R | TCTGGAATGTGGCTATGGACAAC |
| ***Flavonol synthase*** | FLS F | AGTATGGGCAACCACACCAAGAG |
|  | FLS R | GAAGCACAAGAACTGAAGAAGG |
| ***Leucoanthocyanidin reductase*** | LAR F | CAACTTCCCCATTGAAGG |
|  | LAR R | ATTGAAGCACTCATCCAAAG |
| ***Phenylalanine ammonia-lyase*** | PAL F | TTGAAGCTCATGTCTTCCAC |
|  | PAL R | CAAGTTCTCCTCCAAATG |
| ***β-Actin*** | ACT F | TCGTGTTGCCCCAGAAGAG |
|  | ACT R | CACGATTAGCCTTGGGATTCA |
